# CT-based radiomics: predicting early outcomes after percutaneous transluminal renal angioplasty in patients with severe atherosclerotic renal artery stenosis

**DOI:** 10.1186/s42492-023-00152-5

**Published:** 2024-01-12

**Authors:** Jia Fu, Mengjie Fang, Zhiyong Lin, Jianxing Qiu, Min Yang, Jie Tian, Di Dong, Yinghua Zou

**Affiliations:** 1 Department of Interventional Radiology and Vascular Surgery, Peking University First Hospital, Beijing, 100043 China; 2 Department of Radiology, Peking University First Hospital, Beijing, 100043 China; 3 Beijing Advanced Innovation Center for Big Data-Based Precision Medicine, School of Engineering Medicine, Beihang University, Beijing, 100191 China; 4 CAS Key Laboratory of Molecular Imaging, Institute of Automation, Chinese Academy of Sciences, Beijing, 100190 China; 5 School of Artificial Intelligence, University of Chinese Academy of Sciences, Beijing, 100049 China

**Keywords:** Atherosclerotic renal artery stenosis, Percutaneous transluminal renal angioplasty, Computed tomography, Radiomics, Deep learning, Explainability

## Abstract

This study aimed to comprehensively evaluate non-contrast computed tomography (CT)-based radiomics for predicting early outcomes in patients with severe atherosclerotic renal artery stenosis (ARAS) after percutaneous transluminal renal angioplasty (PTRA). A total of 52 patients were retrospectively recruited, and their clinical characteristics and pretreatment CT images were collected. During a median follow-up period of 3.7 mo, 18 patients were confirmed to have benefited from the treatment, defined as a 20% improvement from baseline in the estimated glomerular filtration rate. A deep learning network trained via self-supervised learning was used to enhance the imaging phenotype characteristics. Radiomics features, comprising 116 handcrafted features and 78 deep learning features, were extracted from the affected renal and perirenal adipose regions. More features from the latter were correlated with early outcomes, as determined by univariate analysis, and were visually represented in radiomics heatmaps and volcano plots. After using consensus clustering and the least absolute shrinkage and selection operator method for feature selection, five machine learning models were evaluated. Logistic regression yielded the highest leave-one-out cross-validation accuracy of 0.780 (95%CI: 0.660–0.880) for the renal signature, while the support vector machine achieved 0.865 (95%CI: 0.769–0.942) for the perirenal adipose signature. SHapley Additive exPlanations was used to visually interpret the prediction mechanism, and a histogram feature and a deep learning feature were identified as the most influential factors for the renal signature and perirenal adipose signature, respectively. Multivariate analysis revealed that both signatures served as independent predictive factors. When combined, they achieved an area under the receiver operating characteristic curve of 0.888 (95%CI: 0.784–0.992), indicating that the imaging phenotypes from both regions complemented each other. In conclusion, non-contrast CT-based radiomics can be leveraged to predict the early outcomes of PTRA, thereby assisting in identifying patients with ARAS suitable for this treatment, with perirenal adipose tissue providing added predictive value.

## Introduction

Atherosclerotic renal artery stenosis (ARAS) is defined as the narrowing of the main renal arteries or their branches as a result of atherosclerotic plaques, and is the leading cause of renal artery stenosis [1]. The prevalence of ARAS is approximately 7% among individuals aged 65 years and older and can reach up to 20% among those with diabetes and secondary hypertension [2]. Severe renal artery stenosis may lead to serious complications, such as secondary hypertension, ischemic nephropathy, left ventricular dysfunction, pulmonary edema, and cerebro-cardiovascular events [3]. Therefore, early and prompt treatment is essential for the effective management of the condition.

Several treatment options are available for ARAS, including medical therapy, revascularization, and surgery [4]. Endovascular therapy is often the initial treatment [5]. However, ARAS management is complicated and controversial [4, 5]. On the one hand, three randomized clinical trials (STAR, ASTRAL, and CORAL) indicated that renal artery revascularization did not significantly improve the renal function and prognosis when compared to medical therapy alone [4, 6, 7]. In contrast, recent studies (GSH and KDIGO) have suggested that percutaneous transluminal renal angioplasty (PTRA) may provide potential benefits for selected patients by controlling blood pressure and delaying renal failure [8, 9]. Therefore, it is crucial to identify patient subgroups that may benefit from PTRA before treatment. This will assist clinicians in developing tailored treatment plans for patients with ARAS.

Computed tomography (CT), a noninvasive imaging tool with high resolution, is essential for the clinical diagnosis and treatment planning of patients with ARAS [10]. Previous studies have primarily focused on assessing ARAS based on morphological information such as renal size [9]. Radiomics, which utilizes handcrafted formulas and deep learning networks, extracts high-throughput quantitative features from medical images. By integrating artificial intelligence (AI) methodologies, radiomics also enhances the modeling and analysis processes, offering new insights for radiological diagnosis. It has been proven to objectively evaluate the imaging phenotype characteristics of the renal tissues and lesions [11, 12]. Previous CT-based radiomics research has largely targeted renal tumor diagnosis and constructed predictive models by extracting radiomics features from primary tumors [13, 14] or extra-tumoral invasive tissues [15]. For other types of renal diseases, researchers have explored the feasibility of using radiomics to assist in clinical diagnosis. For example, Shin et al. [16] improved the V-net for precise kidney segmentation and volume measurement, achieving an accuracy comparable to that of human specialists for 50 randomly selected image slices. Amiri et al. [17] utilized a modified Mask R-CNN for kidney segmentation and extracted handcrafted features to train a random forest (RF) model for predicting radiation-induced kidney damage. Patro et al. [18] enhanced network computational efficiency and diagnostic performance for kidney stone detection by introducing a Kronecker product-based convolution. Sudhir Pillai et al. [19] integrated handcrafted features of kidney stone regions using a linear regression model to diagnose fragility. Additionally, as a crucial component of radiomics, research on automatic segmentation algorithms for the kidney and related lesions has been initiated. This is instrumental in standardizing and automating the diagnostic systems. For example, Hsiao et al. [20] developed a segmentation model based on a feature pyramid network that improved the delineation between the kidney and surrounding tissues through multi-scale feature integration. Li et al. [21] proposed a two-stage training strategy applied to five types of deep learning models aimed at the precise segmentation of kidneys and kidney stones.

Drawing on these studies, although there is still a lack of research on and application of CT-based radiomics in renal artery stenosis, this technology holds promise for mining diagnostic and prognostic information on ARAS from abdominal CT images, thereby identifying useful biomarkers for clinical use. Therefore, this retrospective study aimed to evaluate the feasibility of using non-contrast CT-based radiomics features and signatures to predict the early outcomes of PTRA in patients with severe ARAS. Unlike other renal-related CT-based radiomics studies, this study not only extracted features from the affected renal region but also quantified the imaging phenotype of perirenal adipose tissues to gain a more comprehensive understanding of the impact of ARAS. Simultaneously, a pretext task was used to train a deep learning network on unlabeled data, reveal and enhance the intrinsic characteristics of CT images, and explore the benefits of self-supervised learning in radiomics. Additionally, various methods were used to visually demonstrate the potential of radiomics models in predicting the efficacy of endovascular therapy, suggesting that this technique may guide treatment choices for patients with ARAS.

## Methods

### Participants

This single-center retrospective study was approved by the Institutional Review Board of Peking University First Hospital, and the requirement for informed consent was waived. A total of 89 patients treated for severe ARAS at an interventional center between January 2021 and December 2022 were screened for inclusion in the study. The inclusion criteria were as follows: (1) patients aged 40 years or older; (2) patients with ≥ 75% stenosis (including occlusion) on the treatment side of the renal artery, with no definite stenosis in the branches of the renal artery; (3) patients with renal artery stenosis caused by atherosclerosis; (4) patients presenting with clinical symptoms related to renal artery stenosis, such as resistant hypertension; (5) patients who underwent subsequent endovascular treatment; and (6) patients with complete clinical data, including abdominal CT examinations performed within 10 d prior to treatment. The exclusion criteria were as follows: (1) patients with tumors and a history of arterial stenting (*n* = 2); (2) patients without pretreatment CT examinations (*n* = 25); (3) patients with ≥ 1 mo interval between CT scans (*n* = 6); (4) patients with poor CT imaging quality (*n* = 3); and (5) patients lost to follow-up (*n* = 1).

Therefore, 52 patients with the required clinical, radiological, and prognostic data were enrolled. Baseline clinical data, including age, sex, blood pressure, smoking status, serum creatinine (SCr), split glomerular filtration rate (GFR), and estimated GFR (eGFR), were obtained from the medical records [1, 8, 22]. All patients were scanned using a 64-detector row CT scanner (Discovery CT750, USA) in the supine position. The CT scanning settings were as follows: tube voltage, 120 keV; tube current, 200 mAs; interval, 1.25 mm; and slice thickness, 5 mm.

**Fig. 1:**
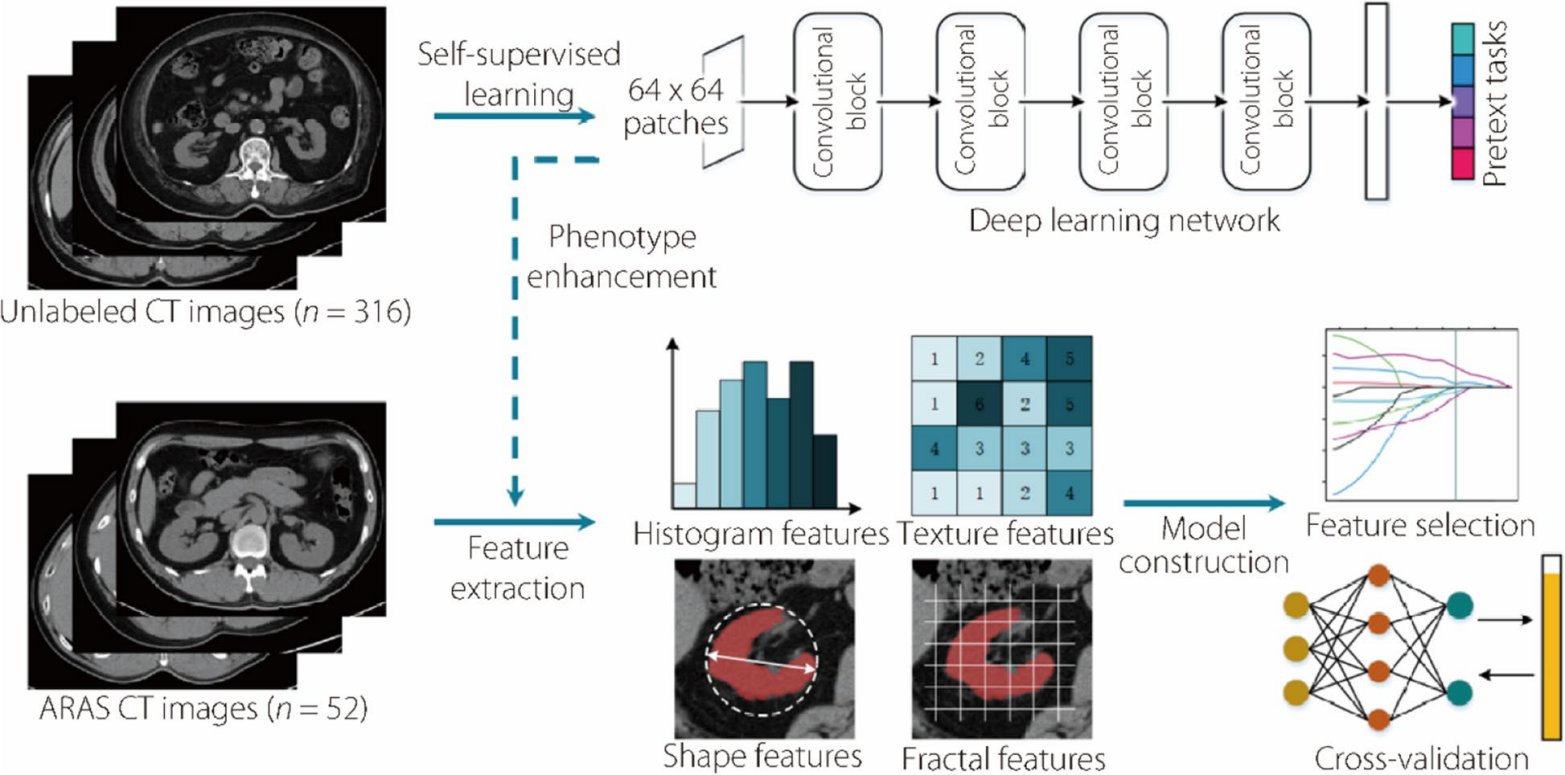
Workflow of radiomics modeling for early outcomes in patients with severe ARAS after PTRA

To enhance the imaging phenotypic characteristics of the renal and adjacent adipose tissues in CT images using deep learning, abdominal non-contrast CT scans were collected from an additional 316 patients to form an unlabeled dataset. The scanning parameters were matched with those of the ARAS dataset. A self-supervised learning approach was used, leveraging the pretext task to allow the network to autonomously learn representation patterns from images. As a result, this dataset did not require the inclusion of patients’ clinical information. The radiomics modeling pipeline is shown in Fig. 1.

### Endovascular treatment and follow-up

This subsection details the endovascular intervention protocols and follow-up rules adopted for patients in the study to clarify the targeted clinical application scenario.

Experienced interventional radiologists performed percutaneous interventions after undergoing artery digital subtraction angiography. Endovascular treatment was administered to patients with severe stenosis and occlusion [1]. A 7 F short sheath was exchanged for arterial access, followed by a 7 F guiding catheter engaging the ostium of the stenotic renal artery, and the stenotic lesion was then crossed with a 0.014-inch guidewire. A rapid exchange balloon, 3–5 mm in diameter, was advanced along the guidewire to predilate the lesion. A balloon-expandable stent was deployed in the stenotic segment via a guiding catheter. Successful intervention was defined as achieving a residual stenosis of < 30% at the completion of angiography.

All patients were followed up for at least 3 mo after PTRA. Blood pressure and renal function were reassessed. For those who could not visit the hospital, doctors conducted telephone and online clinics to obtain and record information. The categorization of patients as benefiting or not was based on renal function, i.e., eGFR [22, 23], as follows: (1) Improvement: ≥ 20% increase in eGFR compared to preoperative levels, which lasted for at least 3 mo; (2) Stable: postoperative change in eGFR < 20%; (3) Exacerbation: ≥ 20% decrease in eGFR compared to preoperative levels. Patients who demonstrated improvement were defined as the benefit group, whereas those who remained stable or experienced worsening were defined as the non-benefit group.

### Image segmentation

The segmentation process was conducted before extracting the radiomics features. To balance efficient clinical use and stable feature extraction, semiautomatic segmentation for the region of interest (ROI) was performed on the axial section where the affected renal tissue showed the largest area, consistent with other renal radiomics studies [24, 25]. This step was performed in all patients using ITK-SNAP (version 3.6.0) by a radiologist with over 10 years of clinical diagnostic experience. Specifically, all segmentations were performed on non-contrast CT and guided by multiplanar reformation. ITK-SNAP offers an “adaptive brush” tool, designed to perform adaptive segmentation within manually designated rectangular areas, based on the intensity variations between different tissues. Initially, the radiologist utilized this tool to obtain a preliminary outline of the ROI, followed by manual adjustments to ensure accurate segmentation while specifically excluding cystic tissues and vessels during this process.

In this study, the affected renal region was segmented as ROI1, while the perirenal adipose region was segmented as ROI2 for radiomics analysis. However, it is noteworthy that segmentation was not possible in two patients because renal parenchymal atrophy blurred the boundaries.

### Hand-crafted feature extraction

For each patient, radiomics features were separately extracted from the renal (ROI1) and perirenal adipose tissues (ROI2) and the association of these features with early outcomes was analyzed.

Before feature extraction, the CT images were preprocessed for standardization, which included aligning the gray-level histograms and adjusting the image pixel size to 0.5 mm through non-linear interpolation. Additionally, smoothing filtering was applied to improve image quality and reduce noise interference. Details, such as boundaries, streaks, and plaques, were enhanced using image differencing. Therefore, in addition to an analysis of the original CT image (X), the post-smoothing (XL) and post-differencing (XH) images were also analyzed. Five categories of handcrafted features were extracted based on these three types of images: shape, histogram, second-order texture, high-order texture, and fractal features, totaling 116.

To improve the comparability of the experimental results, guidelines were adopted from the image biomarker standardization initiative [26] to standardize the feature formulas. Feature extraction was performed using MATLAB (version R2022b).

### Deep learning feature extraction

A self-supervised learning approach was used to train a deep learning network to enhance imaging phenotypes, and deep learning features were subsequently extracted from the enhanced CT images.

Self-supervised learning allows a network to capture profound abstract representations of images using unlabeled data via a pretext task. This approach is particularly suitable for scenarios, such as medical image analysis, where data annotation is expensive and time-consuming. In this study, the network was guided through a pretext task to identify normal abdominal structures using four types of randomly degraded CT images. Specifically, the network was trained to produce a five-class prediction that determined the likelihood of the input image being either normal or altered by one of the specific types of degradation. Image degradation, including blurring, adding noise, morphological distortion, and position displacement, was computer generated and did not require clinical information.

The deep learning network consisted of four convolutional blocks. Each convolutional block had two convolutional layers, a batch normalization layer, a rectified linear unit activation, and a squeeze-and-excitation (SE) block. The convolutional layers used 2 × 2 kernels to generate eight output channels. The SE block enhanced the feature map representations through channel attention mechanisms. Each convolutional block also incorporated a skip connection, employing a 1 × 1 convolutional layer to modify the input feature map before merging it with the block output. This strategy improved the information flow and counteracts the vanishing gradient issue. A 2 × 2 max pooling followed each block for spatial downsampling of the feature map. After the four convolutional blocks, the global average pooling condensed the feature maps into a one-dimensional vector. A Dropout layer reduced the risk of overfitting. Finally, a fully connected layer with five outputs, paired with softmax activation, translated the network output into a probability distribution over five classes, identifying whether the input image was normal and its specific degradation type. Considering the classification-oriented architecture of the network, a categorical cross-entropy loss was employed as the loss function.

During the network training phase, the unlabeled dataset was partitioned into training, validation, and test subsets using a 7:1:2 split. From various CT planes, patches of abdominal tissue of size 64 × 64 were randomly selected, ensuring that there was no patient overlap across the subsets. The learning rate was adjusted dynamically if there was no enhancement in the validation performance after five epochs. Training was halted if no progress was noted over ten successive epochs.

L1-L2 regularization was incorporated within the fully connected layer, which led to sparsification of the model weights. This approach allows significant feature maps to focus on a few nodes. During the subsequent deep learning feature extraction, only features from these information-dense maps with pronounced weights were extracted. In this study, the deep learning features not only included the mean values from the original network node output but also encompassed the statistical and textural attributes of the ROI within the feature maps. These were quantified using the same handcrafted formulas as those employed for the histogram features and second-order texture features described in the previous section.

### Feature selection and model construction

To eliminate differences in dimensions and value ranges among the various radiomics features, z-score normalization was employed. Univariate analysis of each feature was conducted. Features with a *P* value < 0.1 were considered to have potential relevance to early outcomes and were retained for the subsequent selection process.

The Spearman correlation coefficient was used to quantify feature similarity and the partitioning around medoids clustering algorithm was applied to group the features into subsets. Through consensus clustering analysis, both the number of subsets (k) and feature importance within each subset were determined. This involved resampling features at a rate of 50% without replacement, 500 times. The consensus value representing the probability of each feature being grouped with other features in the same subset was recorded. The feature with the highest consensus value in each subset was termed the medoid feature. The appropriate number of subsets (k) were identified by beginning with k = 2 and progressively increasing it, calculating the Spearman correlation between each medoid feature and other features within its subset. This was continued until all correlations were above 0.6, ensuring the retention of all independent imaging phenotypes while eliminating redundancy.

Subsequently, the least absolute shrinkage and selection operator (LASSO) algorithm paired with logistic regression (LR) was employed to refine the feature selection. LASSO utilizes L1 regularization to penalize less influential features, thereby progressively identifying the most crucial feature combination for prediction. Finally, five machine learning models were built and assessed: LR, artificial neural network, K-nearest neighbor, RF, and support vector machine (SVM). A 10-fold cross-validation and grid search was employed to finalize the input features and determine the optimal hyperparameters for each model. Subsequently, the best model for predicting early outcomes in patients with severe ARAS was determined based on the average accuracy from leave-one-out cross-validation, which was used for the construction of the renal signature and the perirenal adipose signature.

### Statistical analysis

The Shapiro-Wilk test was used to assess the normality of the features. For normally distributed features, an independent samples *t*-test was used to determine the differences between the benefit and non-benefit groups. Otherwise, the Mann-Whitney U test was used. A *P* value of < 0.05 was deemed statistically significant. Volcano plots and radiomics heatmaps were used to analyze the relationship between renal and perirenal adipose CT radiomics features and early outcomes in patients with ARAS. In addition, principal component analysis (PCA) was employed to visually represent the inherent patterns within the feature sets.

The predictive power of the features and models for early outcomes was evaluated using confusion matrix, threshold analysis, and the receiver operating characteristic (ROC) curve. The area under the ROC curve (AUC) was calculated. Owing to the limited sample size of patients with severe ARAS, leave-one-out cross-validation was used to ensure a more objective assessment of the applicability and generalizability of the radiomics signatures. By testing each data sample individually and training on the remaining data, this method provides a nearly unbiased error estimate for the model within the dataset. Additionally, SHapley Additive exPlanations (SHAP) analysis was used [27] to interpret the decision-making rationale of radiomics signatures and to understand the importance and roles of each feature within them.

R software (version 4.2.1) and Python (version 3.9.12) were used for data analysis, modeling, and performance evaluation in this study.

## Results

### Clinical characteristics

Based on the follow-up results, 18 patients were categorized into the benefit group and the remaining 34 into the non-benefit group. Table 1 summarizes their clinical characteristics. Univariate analysis revealed no significant differences in the clinical characteristics between the two groups. A larger sample size may be more conducive for identifying predictive indicators. The most promising indicator was the pretreatment eGFR: 77.8 ± 31.4 in the benefit group and 63.8 ± 26.7 in the non-benefit group.

**Table 1:** Comparison of the clinical characteristics of patients between benefit and non-benefit groups

Item	Benefit group (*n* = 18)	Non-benefit group (*n* = 34)	*P* value
Stenosis type (*n* [%])			1.000
Unilateral	16 (88.89)	31 (91.18)	
Bilateral	2 (11.11)	3 (8.82)	
Age (year, mean ± SD)	59.20 ± 16.40	59.50 ± 15.20	0.943
Gender (*n* [%])			0.727
Female	3 (16.67)	8 (23.53)	
Male	15 (83.33)	26 (76.47)	
Body mass index (kg/m^2^, mean ± SD)	25.10 ± 4.26	24.70 ± 2.46	0.683
Diabetes (*n *[%])			0.543
No	18 (100)	31 (91.18)	
Yes	0	3 (8.82)	
Coronary heart disease (*n *[%])			0.289
No	14 (77.78)	20 (58.82)	
Yes	4 (22.22)	14 (41.18)	
Smoking (*n *[%])			1.000
No	12 (66.67)	24 (70.59)	
Yes	6 (33.33)	10 (29.41)	
Preoperative ipsilateral GFR (ml/min, mean ± SD)	24.60 ± 9.72	21.70 ± 11.70	0.349
Preoperative eGFR (ml/min/1.73 m^2^, mean ± SD)	63.80 ± 26.70	77.80 ± 31.40	0.100
Preoperative SCr (µmol/L, mean ± SD)	140 ± 156	96.70 ± 25.50	0.258
Maximum systolic blood pressure (mmHg, mean ± SD)	169 ± 21.30	179 ± 28.20	0.180
Maximum diastolic blood pressure (mmHg, mean ± SD)	98.80 ± 18.60	98.70 ± 19.50	0.994
Hemoglobin (g/L, mean ± SD)	127 ± 20.00	130 ± 14.40	0.486
Potassium (mmol/L, mean ± SD)	3.95 ± 0.38	3.88 ± 0.56	0.568
Sodium (mmol/L, mean ± SD)	140 ± 2.05	140 ± 1.72	0.546

### Renal and perirenal adipose CT radiomics features

**Fig. 2:**
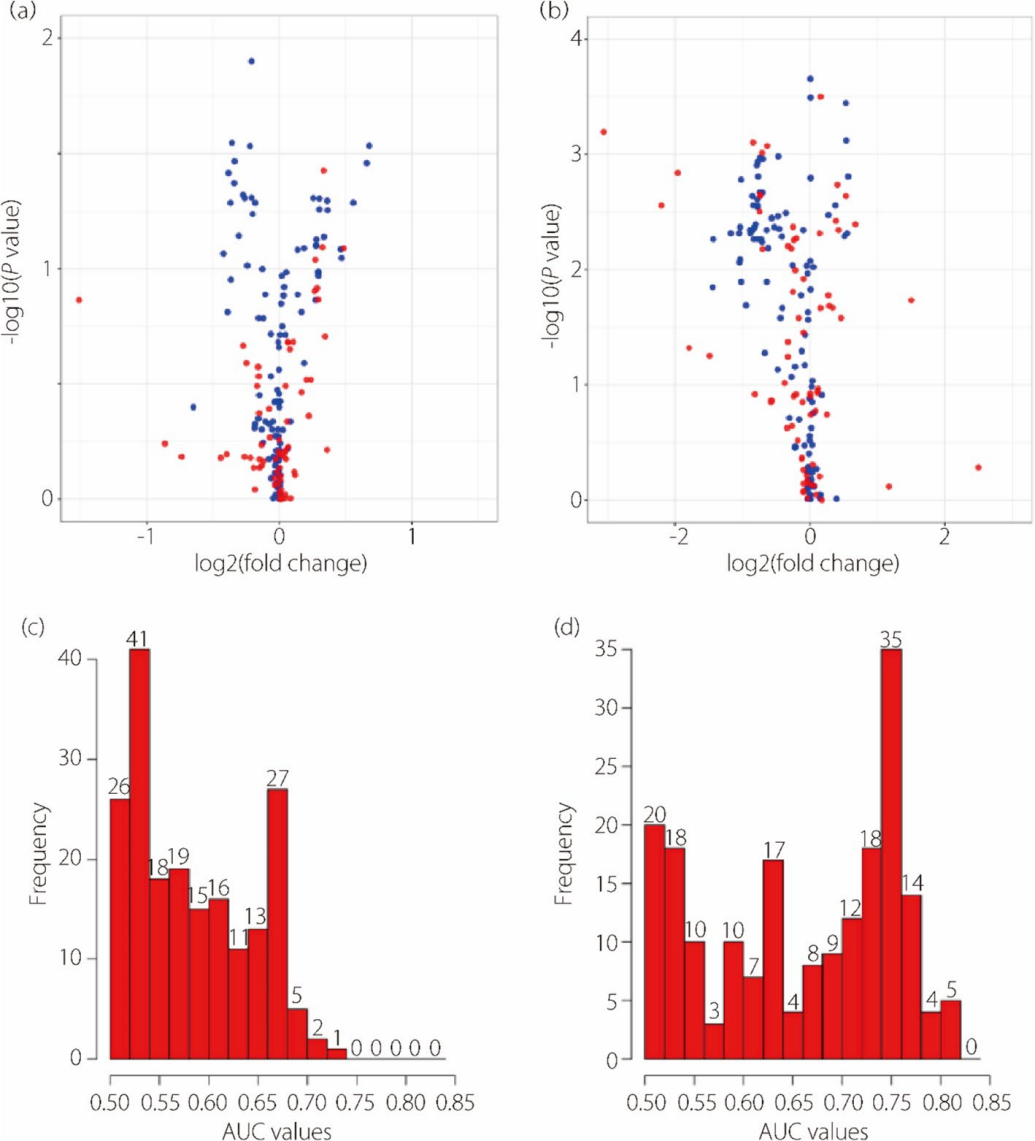
Association of radiomics features with early outcomes. (**a**) Volcano plot for renal features; (**b**) Volcano plot for perirenal adipose features; (**c**) Histogram of AUC for renal features; (**d**) Histogram of AUC for perirenal adipose features. In the volcano plot, blue dots represent handcrafted features, while red dots represent deep learning features

From non-contrast CT images, 194 radiomics features in the affected renal and perirenal adipose regions were extracted. Many features in both regions correlated with early outcomes. Notably, the perirenal adipose region had a larger number of significant features with *P* values < 0.05 (*n* = 101 *vs. n* = 14), as shown in the volcano plots in Fig. 2a and b. This plot also highlights the pronounced differences in the feature values between the two groups for certain highly significant deep learning features. Additionally, in Fig. 2c and d, the histogram of the AUCs for the perirenal adipose features shows a concentration in the high-value area on the right side.

**Fig. 3:**
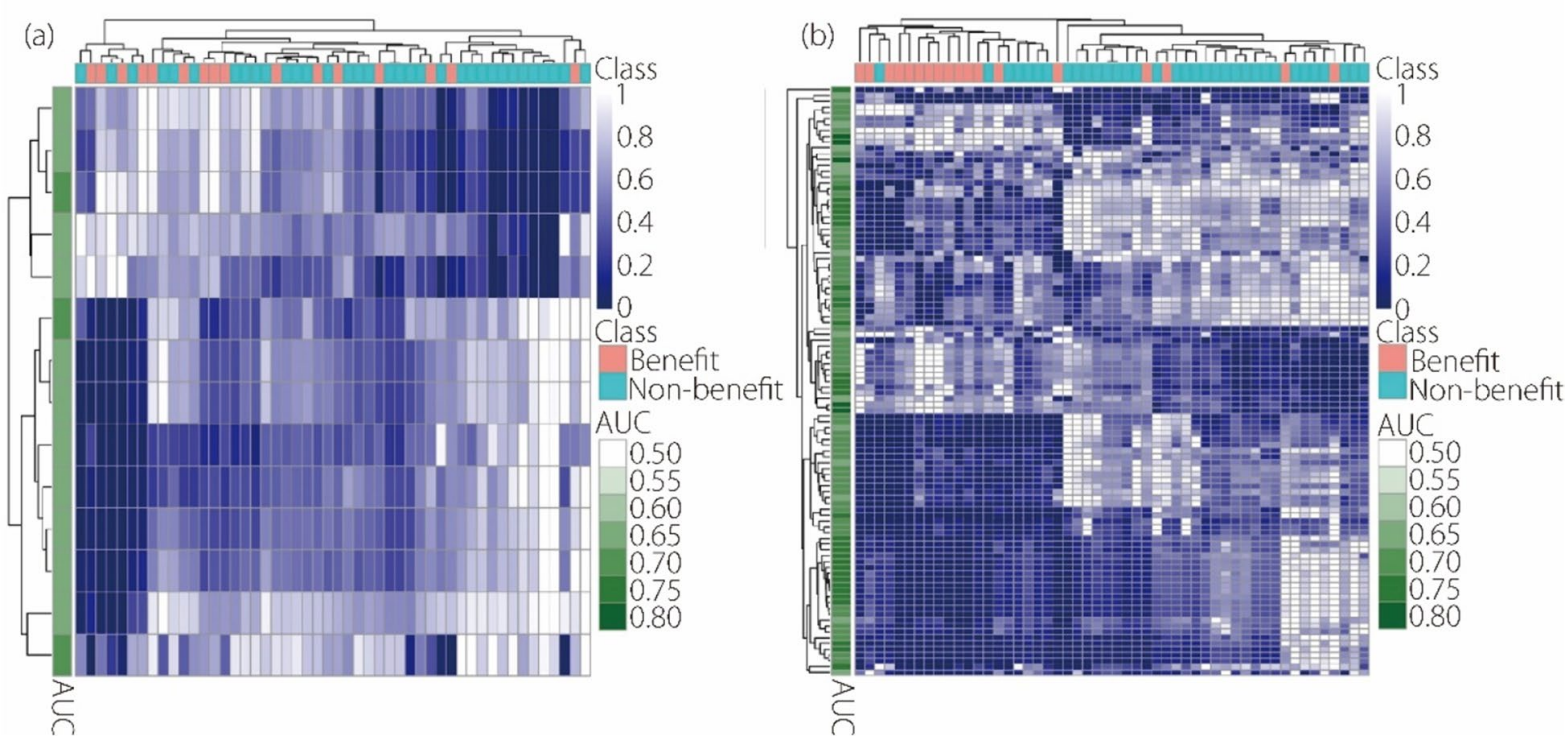
Radiomics heatmaps for renal features (**a**) and perirenal adipose features (**b**). The horizontal axis represents individual patients, and the vertical axis represents radiomics features

Furthermore, unsupervised clustering was performed for both the patients and the above significant features. This was visually represented as radiomics heatmaps (Fig. 3) to contrast the feature value distribution between the benefit and non-benefit groups. The heatmaps of both regions showed clusters of patients with analogous feature expressions, indicating feature subsets that correlated with early outcomes. Notably, the clustering tree derived from perirenal adipose features better preserved patient distance relationships (cophenetic distances: 0.791 *vs.* 0.720) and had a higher correlation with early outcomes (adjusted Rand index: 0.363* vs.* 0.167).

### Feature selection

**Fig. 4:**
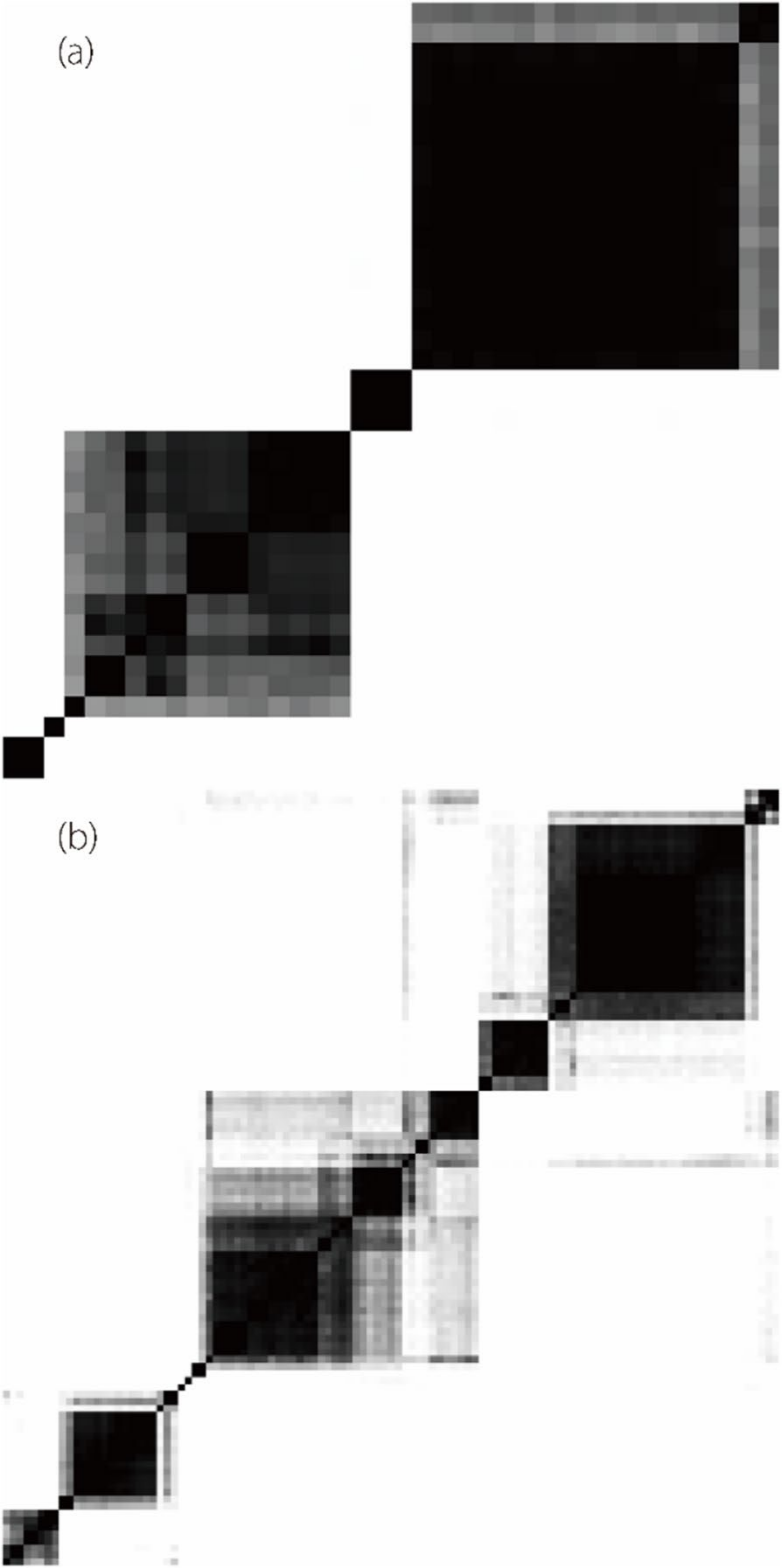
Heatmaps of consensus values for renal features (**a**) and perirenal adipose features (**b**)

Consensus clustering analysis based on the Spearman correlation coefficient was used to identify the optimal number of feature clusters representing independent imaging phenotypes. This method resulted in 5 clusters for renal features and 11 for perirenal adipose features (Fig. 4). Because each cluster displayed consistency in its features, only the medoid feature was retained from each, which is detailed in Table 2.

**Table 2:** Medoid features and feature selection result

Feature type	Feature name	*P* value	AUC	Selected by LASSO	Signature input
Renal feature	XH_H_uniformity	0.052	0.673 (0.502–0.843)	√	√
	XL_H_mean_absulute_deviation	0.034	0.675 (0.519–0.830)	√	
	XH_GLCM_correlation	0.013	0.722 (0.573–0.870)	√	√
	XL_GLRLM_LRHGLE	0.090	0.651 (0.491–0.811)		
	D1_GLCM_correlation	0.096	0.675 (0.500-0.849)	√	√
Perirenal adipose feature	XL_H_uniformity	0.005	0.737 (0.578–0.896)		
	X_GLCM_contrast	0.005	0.740 (0.592–0.889)	√	
	XL_H_krutosis	0.053	0.665 (0.489–0.841)		
	XL_H_standard_deviation	0.005	0.740 (0.583–0.898)		
	XH_H_mean	< 0.001	0.815 (0.688–0.941)	√	
	XH_GLCM_cluster_shade	< 0.001	0.815 (0.689–0.942)	√	√
	D5_GLCM_homogeneity2	0.006	0.748 (0.595–0.902)	√	√
	XH_GLCM_correlation	0.070	0.618 (0.440–0.795)	√	
	D1_H_maximum	0.012	0.714 (0.563–0.865)		
	D5_GLCM_entropy	0.026	0.685 (0.526–0.843)		
	D1_GLCM_energy	0.004	0.745 (0.585–0.906)	√	

*Dx* Xth deep learning feature map, *H* Histogram feature, *GLCM* Gray-level co-occurrence matrix-based feature, *GLRLM* Gray-level run length matrix feature, *LRHGLE* Low run high gray-level emphasis

**Fig. 5:**
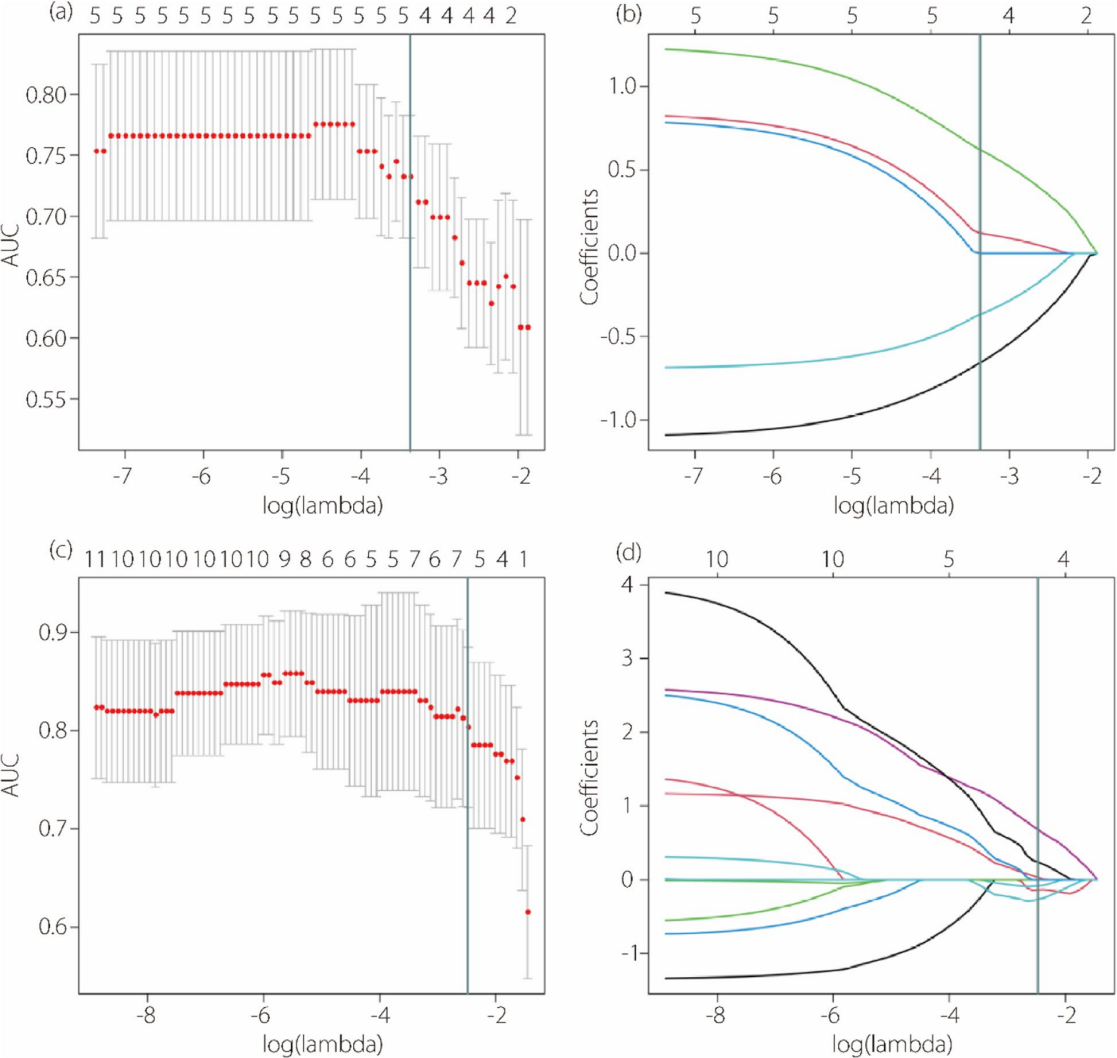
LASSO feature selection process for renal features (**a**, **b**) and perirenal adipose features (**c**, **d**)

To further refine the feature selection, LASSO combined with LR was employed to search for the key feature combination. The regularization coefficients were iteratively adjusted, with the one that yielded the highest cross-validation AUC selected. The step-by-step LASSO selection for renal and perirenal adipose features is shown in Fig. 5. From this process, four renal features and six perirenal adipose features were retained, which are presented in Table 2.

**Fig. 6:**
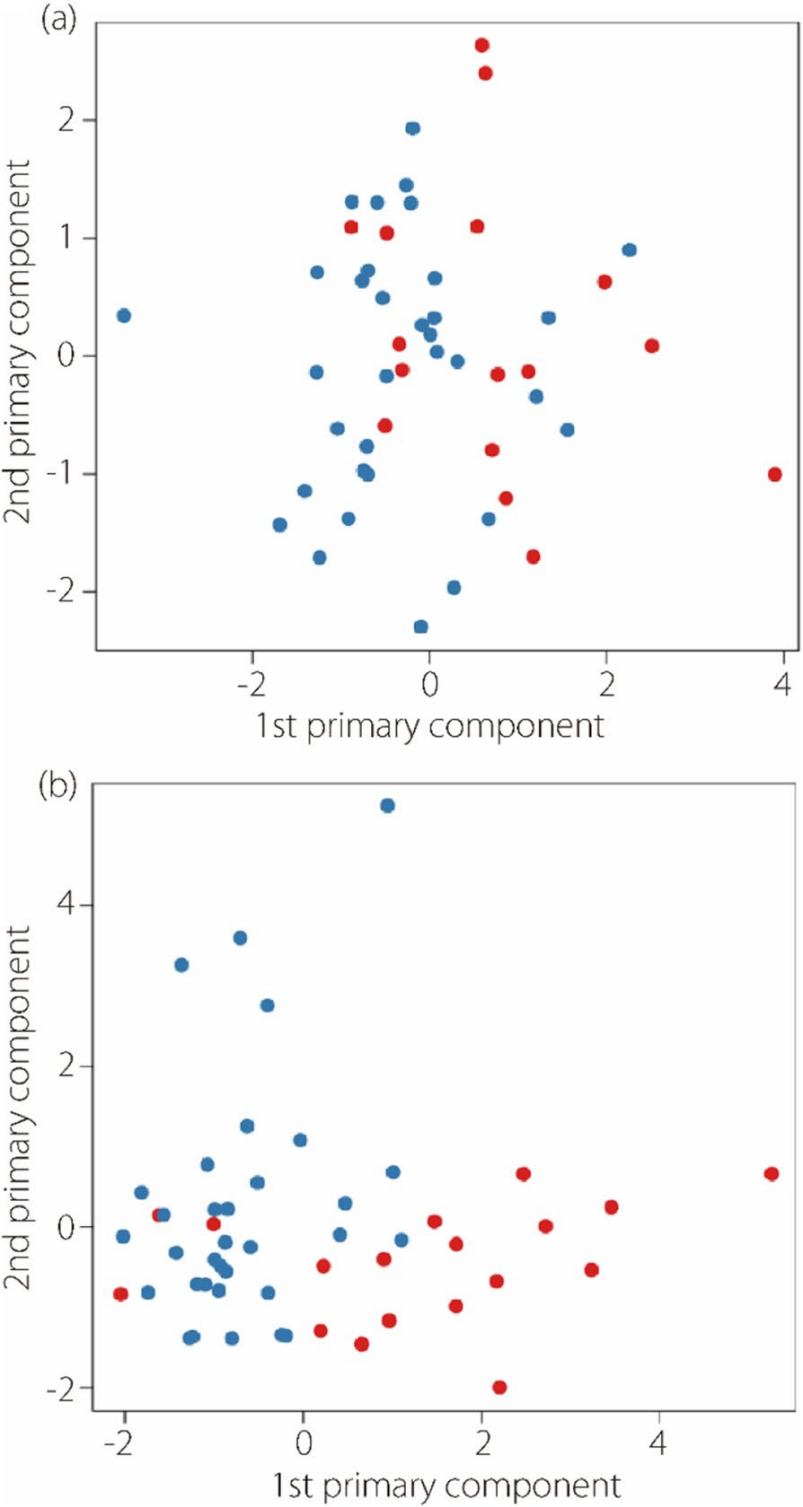
The two primary components of the selected renal features (**a**) and perirenal adipose features (**b**). Red dots represent the benefit group, while blue dots represent the non-benefit group

PCA visualization (Fig. 6) showed that the primary components of the selected perirenal adipose features effectively differentiated between benefit and non-benefit. Conversely, although the primary components of the renal features were effective, there was noticeable overlap between the two groups. This suggests that the retained renal features contain information that is either irrelevant to treatment outcomes or has ambiguous associations.

### Model construction and performance analysis

**Fig. 7:**
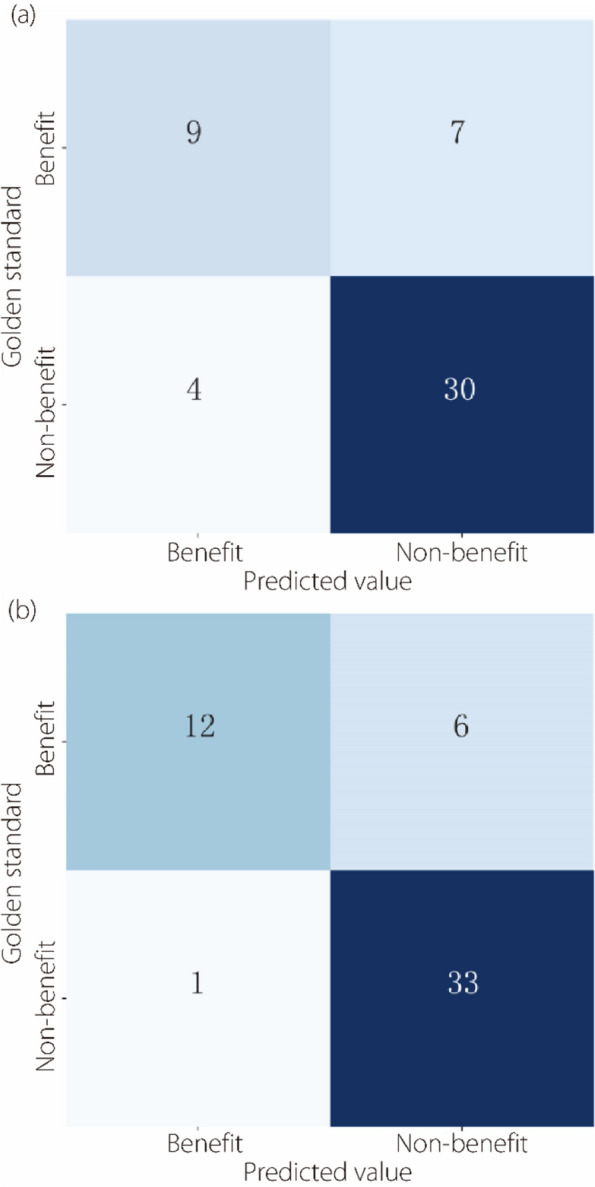
Confusion matrices of the leave-one-out cross-validation results. (**a**) LR with renal features as input; (**b**) SVM with perirenal adipose features as input

Five machine learning models were trained and their performance assessed. During leave-one-out cross-validation, LR achieved the highest accuracy of 0.780 (95%CI: 0.660–0.880) with renal features (Fig. 7). Meanwhile, using perirenal adipose features, the radial basis function-kernel SVM yielded an accuracy of 0.865 (95%CI: 0.769–0.942). Accordingly, a renal signature was developed using LR with three features, and a perirenal adipose signature using SVM with two features (Table 2). The AUC of the perirenal adipose signature was 0.879 (95%CI: 0.759–1.000), which was superior to that of the renal signature, 0.829 (95%CI: 0.691–0.967).

**Fig. 8:**
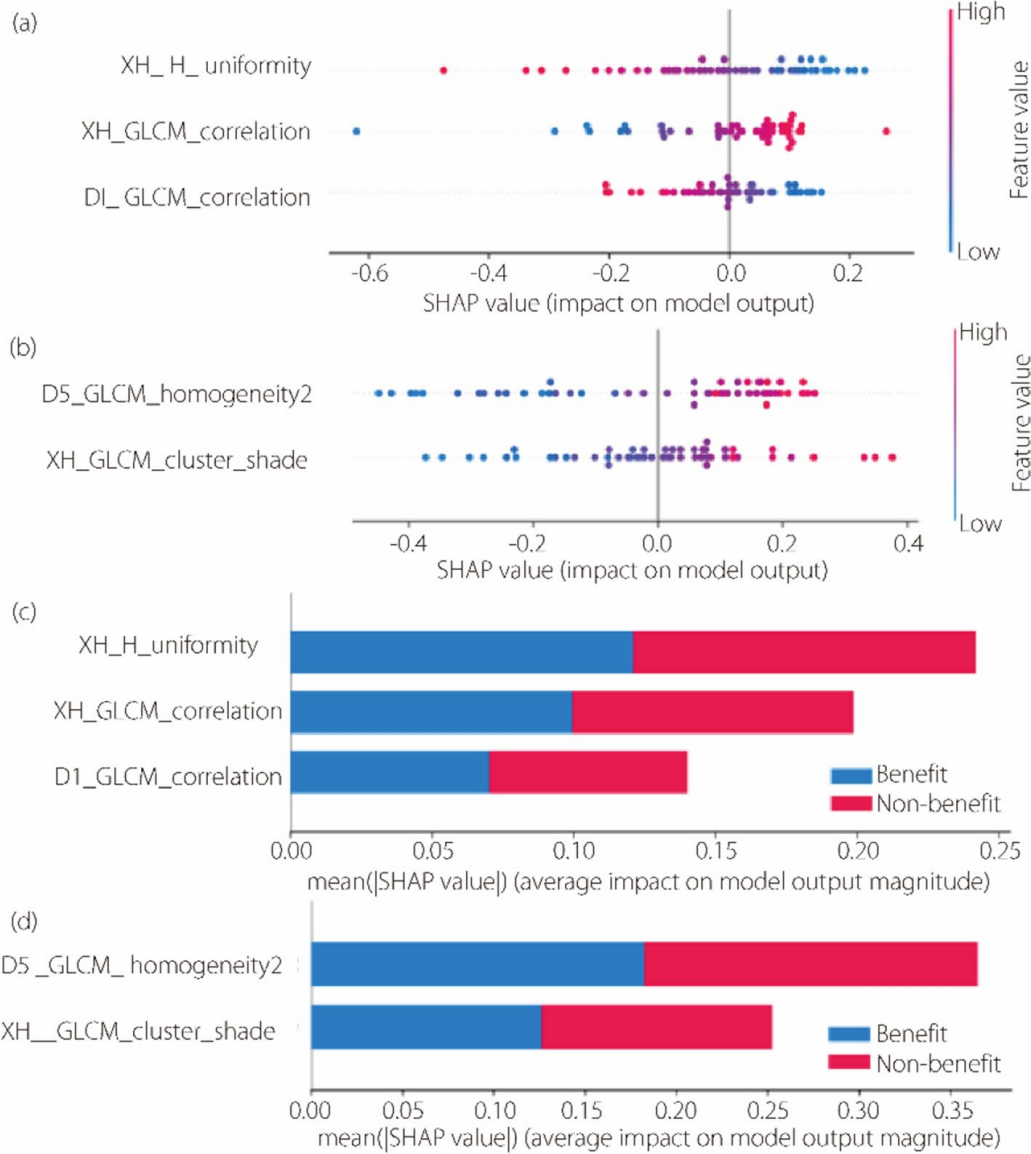
SHAP summary plots of the renal signature (**a**, **c**) and perirenal adipose signature (**b**, **d**). **a**, **b** The plots illustrate the Shapley value for each feature in every patient; **c**,** d** The plots illustrate the average of the absolute SHAP values for each feature

The SHAP kernel explainer was used to evaluate feature attributions by quantifying the individual impact of each feature on the predictions of different medical signatures. SHAP summary plots were used to visually depict the distribution of feature importance for each signature’s output, with the features sorted vertically based on their global importance. The SHAP values for each feature from various patients are represented by horizontal dots in Fig. 8a and b. Additionally, Fig. 8c and d illustrates the average absolute SHAP value for each feature. The results indicate that a deep learning feature has the most significant impact on decision-making for the perirenal adipose signature, whereas for the renal signature, a histogram feature plays the most crucial role.

**Fig. 9:**
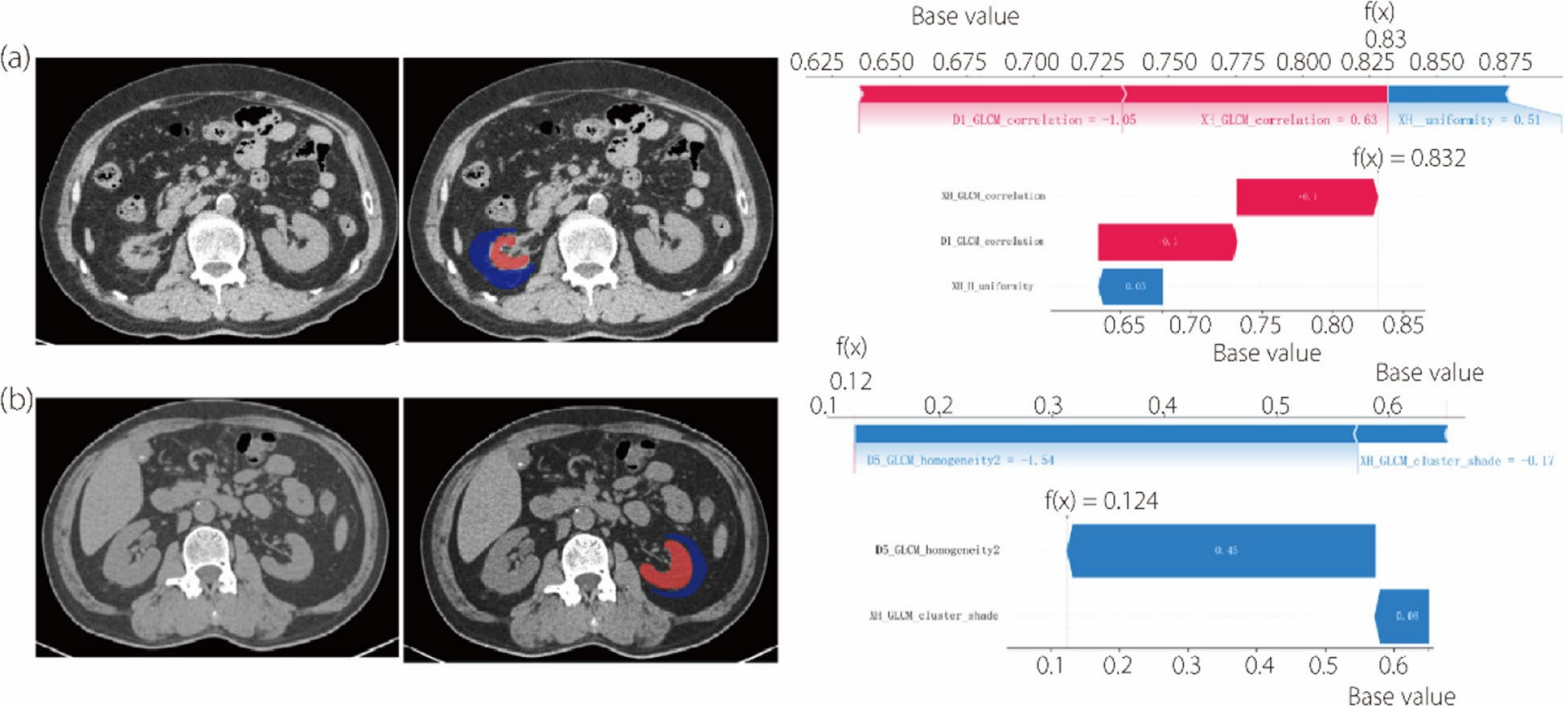
SHAP force plots illustrating the discrimination of the early outcomes for two patients based on radiomics signatures. (**a**) The renal signature identifies a patient who did not benefit from PTRA; (**b**) The perirenal adipose signature identifies a patient who benefited from PTRA

Then, the SHAP force plot was used to explain how individual features influenced the prediction of signatures for a single patient, displaying the direction and magnitude of each feature’s contribution using blue or red arrows. As shown in Fig. 9, when the arrows of each feature in the signature are overlaid, if the SHAP value is lower than the base value, the signature predicts that the patient will benefit from PTRA. Conversely, this suggests a significant risk of the patient not benefiting.

**Fig. 10:**
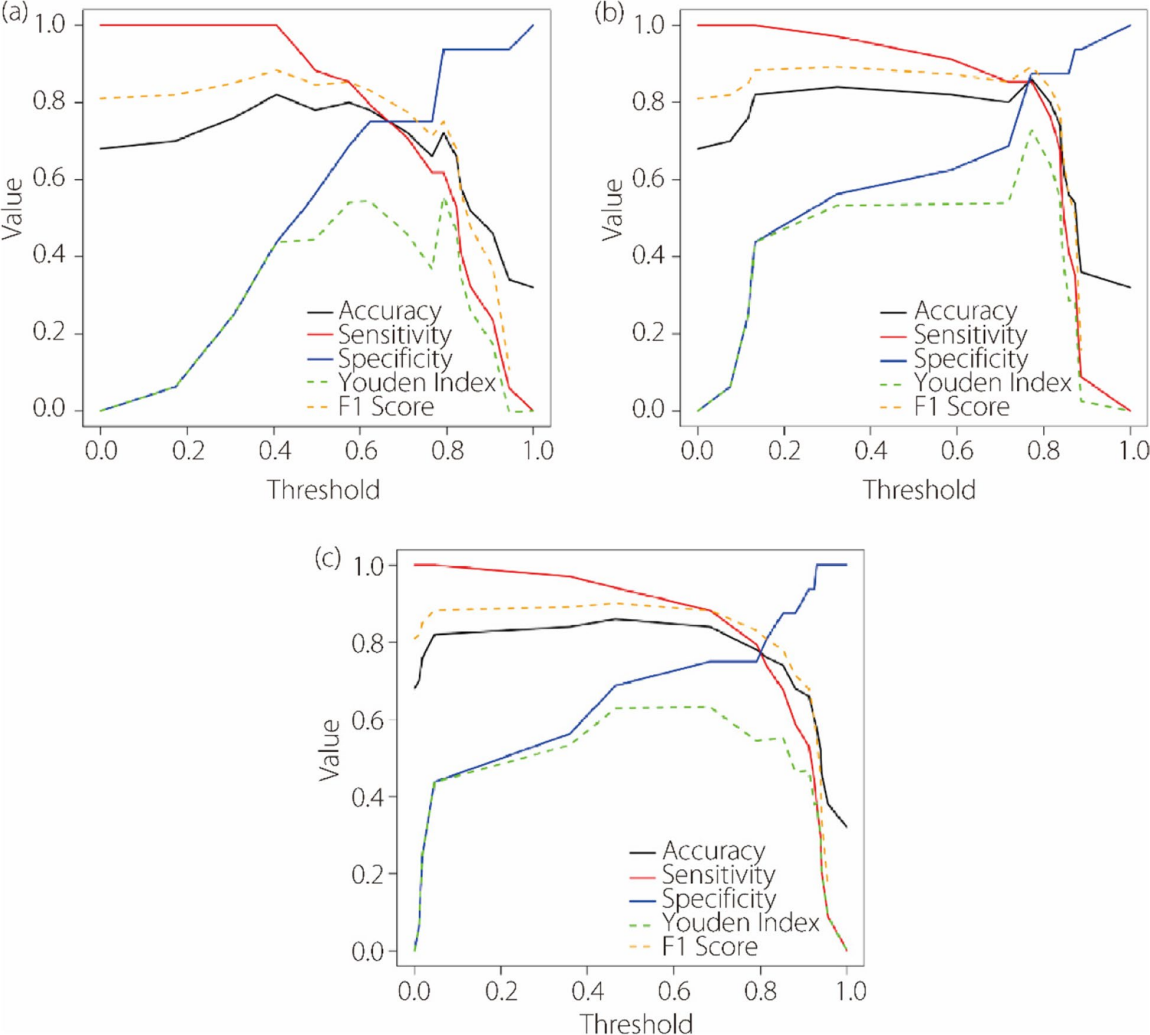
Threshold analysis for accuracy, sensitivity, specificity, Youden index, and F1 score for the renal signature (**a**), perirenal adipose signature (**b**) and combined model (**c**)

To investigate the synergy between renal and perirenal adipose signatures, LR was used to integrate them. Each signature emerged as a significant independent predictive factor in multivariate analysis, with *P* values < 0.05 (renal signature at 0.033 and perirenal adipose signature at 0.005). No multicollinearity was detected between the signatures, as indicated by variance inflation factors below 5. As shown in Fig. 10, a threshold analysis was used to measure the performance metrics of each predictive model as the classification threshold varied from 0 to 1. The ROC curves further revealed that the combined model yielded a superior AUC of 0.888 (95%CI: 0.784–0.992). This implies that merging insights from both regions may improve prediction accuracy.

## Discussion

This study evaluated the predictive performance of non-contrast CT-based radiomics for PTRA treatment outcomes in patients with severe ARAS. The results suggest that through the in-depth quantification and integration of CT imaging phenotypes, radiomics has the potential to preoperatively identify patients who are likely to benefit from PTRA. Considering that the prediction process can be seamlessly integrated into the regular ARAS diagnostic workflow and yield results within minutes, radiomics is expected to assist clinicians in determining the most suitable candidates for PTRA.

Renal artery revascularization, primarily via PTRA, is currently one of the main therapeutic approaches for patients with ARAS. However, its efficacy remains unclear. Given the diverse and intricate nature of ARAS, it is essential to identify the specific phenotypes that could benefit most from PTRA. The KDIGO Consensus recommends high-grade stenosis as the primary indication for revascularization [9], supported by a recent prospective cohort study [8]. Therefore, it was hypothesized that patients with severe stenosis would be more likely to benefit from PTRA. Accordingly, the experiments in this study focused on this ARAS subset to explore the feasibility of radiomics in differentiating patients with significant renal function improvements.

Several clinical characteristics, such as GFR and SCr [28, 29], have been shown to correlate with post-PTRA renal function improvement. However, in this study, no statistically significant differences in these factors were observed between the benefit and non-benefit groups. This could be due to the complex recovery mechanisms of renal function, which can be influenced by various factors including ischemia [30]. Moreover, there are differences in the structure and size of the patient cohorts between previous studies and the present study. With a focus on clinical utility, this study specifically targeted patients with severe stenosis who were more likely to benefit from renal revascularization. Therefore, the discriminative efficacy of these factors may have been suppressed in the ARAS subset. Additionally, diabetes has been reported to significantly lower the chances of renal function improvement in patients with ARAS after stenting with optimal medical therapy [25]. However, because only three patients with diabetes participated in the study, the impact of this factor could not be validated.

To our knowledge, no studies have used AI to predict outcomes for patients with ARAS by analyzing preoperative medical images. Few studies have explored the relationship between preoperative magnetic resonance imaging metrics and improvements in renal function [31]. In this study, deep learning radiomics signatures was developed from routine non-contrast CT scans. Notably, the analysis was not limited to the affected renal region but also segmented the perirenal adipose tissue as an added ROI for feature extraction. The renal parenchyma directly reflects renal artery stenosis and ischemia, whereas perirenal adipose can indicate the accumulation of perirenal inflammatory factors [32]. Therefore, it was hypothesized that preoperative imaging phenotypes of these two regions would provide valuable information for predicting the efficacy of PTRA. This was confirmed by the experimental findings, which showed image patterns in both regions that possessed predictive potential. Furthermore, the radiomics signature derived from the perirenal adipose region showed greater predictive capacity than that derived from the renal region.

AI-based medical image analysis has always been constrained by the lack of high-quality datasets. By predicting the attributes of the input data itself (e.g., through a pretext task), self-supervised learning allows models to capture the intrinsic attributes and patterns of the input data, generating more diverse and generalizable feature representations. This enables models to be effectively trained and applied in areas with scarce data, such as medicine [33]. In this study, a set of unlabeled abdominal CT scans were collected and self-supervised learning was conducted on a deep learning network through a pretext task, training to derive a deep learning feature extractor. The extracted high-dimensional features can more thoroughly reveal potential biological patterns in the images, thereby improving the predictive accuracy of the radiomics signature [34]. The constructed renal and perirenal adipose signatures incorporated deep learning features, indicating that these features play a crucial complementary role to handcrafted features in capturing information related to pathological changes and treatment outcomes.

In addition to using a self-supervised learning-based network training scheme, multiple techniques were adopted in this study to address the challenge of a limited sample size, ensuring the enhanced reliability and thoroughness of the findings. Through visualization based on volcano plots and radiomics heatmaps, we were able to intuitively observe the correlations between features and their associations with PTRA outcomes. Both renal and perirenal adipose features were discovered to contain elements indicative of patient response to treatment, showing the potential for constructing predictive models. Leveraging unsupervised clustering, we unveiled latent patterns in radiomics features, filtering out the most representative candidate feature set without the risk of overfitting (comprising 5 renal features and 11 perirenal adipose features). Subsequently, these features were integrated using various machine learning methods. Through a series of cross-validations, it was determined that LR and SVM exhibited stable performances, making them suitable for constructing the radiomics signatures. To gain deeper insights into the decision-making process of signatures, SHAP values were employed for explainability analysis. This approach evaluates the contribution of input features to prediction and showed how they positively or negatively impact prediction outcomes, thereby enhancing the transparency and trustworthiness of decisions. This multifaceted analysis was conducted to comprehensively investigate the inherent information within the data, offering robust references for clinical application and future research.

This study has several limitations. First, eGFR and SCr are the prevalent indicators used in current clinical research on renal artery stenosis for assessing patient renal function. In this study, a post-treatment eGFR improvement exceeding 20% was considered a benefit of PTRA. Considering that SCr is also a commonly used baseline indicator in the diagnosis and treatment of renal artery stenosis, an evaluation of how the key features and signatures of this study correlate with SCr may amplify the clinical relevance of the findings. Second, given that this study focused on patients with severe ARAS, a rigorous data inclusion protocol was implemented, resulting in a relatively small sample size. This limitation prevented the allocation of a separate test set. Consequently, to apply radiomics to this unique and critical clinical domain and explore its applicability, this study employed various techniques, including self-supervised learning, cross-validation, visualization, and model interpretation. Future efforts will require more profound validation via larger-scale multicenter cohorts to facilitate the integration of radiomics into clinical practice. Finally, current large language models [35] and medical foundation models [36] have been proven to effectively learn domain-general knowledge, outperforming previous AI techniques across various tasks. This evolution presents new avenues for the scaled deployment of AI. By developing a renal disease foundational model using general renal disease data and requiring only a small number of specific samples for fine-tuning, specialized models can be effectively deployed for downstream tasks. This approach promises improved predictive accuracy.

## Conclusions

In conclusion, CT-based radiomics features show a robust correlation with early outcomes in patients with ARAS. Radiomics signatures offer the potential to identify patients likely to benefit from PTRA prior to treatment and guide the selection of suitable therapeutic approaches.

## Data Availability

Original imaging and clinical data are not publicly available because they contain private patient health information. Data supporting the findings of the present study are available upon reasonable request.

## Abbreviations

ARAS: Atherosclerotic renal artery stenosis
PTRA: Percutaneous transluminal renal angioplasty
CT: Computed tomography
AI: Artificial intelligence
SCr: Serum creatinine
GFR: Glomerular filtration rate
eGFR: Estimated GFR
ROI: Region of interest
ReLU: Rectified linear unit
SE: Squeeze-and-excitation
LASSO: Least absolute shrinkage and selection operator
LR: Logistic regression
SVM: Support vector machine
PCA: Principal component analysis
ROC: Receiver operating characteristic
AUC: Area under ROC curve
SHAP: SHapley Additive exPlanations
RF: Random forest
X: Original CT image
XL: Post-smoothing image
XH: Post-differencing image
Dx: Xth deep learning feature map
H: Histogram feature
GLCM: Gray-level co-occurrence matrix-based feature
GLRLM: Gray-level run length matrix feature
LRHGLE: Low run high gray-level emphasis

## References

[1] Safian RD (2021). Renal artery stenosis. Prog Cardiovasc Dis.

[2] Mishima E, Suzuki T, Ito S (2020). Selection of patients for angioplasty for treatment of atherosclerotic renovascular disease: predicting responsive patients. Am J Hypertens.

[3] Triantis G, Chalikias GK, Ioannidis E, Dagre A, Tziakas DN (2022). Renal artery revascularization is a controversial treatment strategy for renal artery stenosis: a case series and a brief review of the current literature. Hellenic J Cardiol.

[4] Prince M, Tafur JD, White CJ (2019). When and how should we revascularize patients with atherosclerotic renal artery stenosis?. JACC Cardiovasc Interv.

[5] Mangiacapra F, Trana C, Sarno G, Davidavicius G, Protasiewicz M, Muller O (2010). Translesional pressure gradients to predict blood pressure response after renal artery stenting in patients with renovascular hypertension. Circ Cardiovasc Interv.

[6] Schoepe R, McQuillan S, Valsan D, Teehan G, Islam MS (2017). Atherosclerotic renal artery stenosis. Hypertension: from basic research to clinical practice. Advances in experimental medicine and biology.

[7] Bax L, Woittiez AJJ, Kouwenberg HJ, Mali WPTM, Buskens E, Beek FJA (2009). Stent placement in patients with atherosclerotic renal artery stenosis and impaired renal function: a randomized trial. Ann Intern Med.

[8] Reinhard M, Schousboe K, Andersen UB, Buus NH, Rantanen JM, Bech JN (2022). Renal artery stenting in consecutive high-risk patients with atherosclerotic renovascular disease: a prospective 2-center cohort study. J Am Heart Assoc.

[9] Hicks CW, Clark TWI, Cooper CJ, De Bhailís ÁM, De Carlo M, Green D (2022). Atherosclerotic renovascular disease: A KDIGO (kidney disease: improving global outcomes) controversies conference. Am J Kidney Dis.

[10] Castelli PK, Dillman JR, Smith EA, Vellody R, Cho K, Stanley JC (2013). Imaging of renin-mediated hypertension in children. AJR Am J Roentgenol.

[11] Ursprung S, Beer L, Bruining A, Woitek R, Stewart GD, Gallagher FA (2020). Radiomics of computed tomography and magnetic resonance imaging in renal cell carcinoma-a systematic review and meta-analysis. Eur Radiol.

[12] Alnazer I, Bourdon P, Urruty T, Falou O, Khalil M, Shahin A (2021). Recent advances in medical image processing for the evaluation of chronic kidney disease. Med Image Anal.

[13] Zhou HY, Mao HX, Dong D, Fang MJ, Gu DS, Liu XL (2020). Development and external validation of radiomics approach for nuclear grading in clear cell renal cell carcinoma. Ann Surg Oncol.

[14] Li XL, Ma QL, Nie P, Zheng YM, Dong C, Xu WJ (2022). A CT-based radiomics nomogram for differentiation of renal oncocytoma and chromophobe renal cell carcinoma with a central scar-matched study. Br J Radiol.

[15] Zhao X, Yan Y, Xie WF, Zhao LT, Zhang SD, Liu JG et al (2023) The application of CT radiomics in the diagnosis of vein wall invasion in patients with renal cell carcinoma combined with tumor thrombus. The Oncologist, 2023, oyad243. 10.1093/oncolo/oyad243 10.1093/oncolo/oyad243 PMC10836321 37672362

[16] Shin TY, Kim H, Lee JH, Choi JS, Min HS, Cho H (2020). Expert-level segmentation using deep learning for volumetry of polycystic kidney and liver. Investig Clin Urol.

[17] Amiri S, Akbarabadi M, Abdolali F, Nikoofar A, Esfahani AJ, Cheraghi S (2021). Radiomics analysis on CT images for prediction of radiation-induced kidney damage by machine learning models. Comput Biol Med.

[18] Patro KK, Allam JP, Neelapu BC, Tadeusiewicz R, Acharya UR, Hammad M (2023). Application of Kronecker convolutions in deep learning technique for automated detection of kidney stones with coronal CT images. Inform Sci.

[19] Sudhir Pillai P, Hsieh SS, Vercnocke AJ, Potretzke AM, Koo K, Mccollough CH (2023). *In vivo* prediction of kidney stone fragility using radiomics-based regression models. J Endourol.

[20] Hsiao CH, Lin PC, Chung LA, Lin FYS, Yang FJ, Yang SY (2022). A deep learning-based precision and automatic kidney segmentation system using efficient feature pyramid networks in computed tomography images. Comput Meth Prog Bio.

[21] Li D, Xiao CD, Liu Y, Chen Z, Hassan H, Su LYL (2022). Deep segmentation networks for segmenting kidneys and detecting kidney stones in unenhanced abdominal CT images. Diagnostics.

[22] Levey AS, Stevens LA, Schmid CH, Zhang YP, Castro AF, Feldman HI (2009). A new equation to estimate glomerular filtration rate. Ann Intern Med.

[23] Modrall JG, Zhu H, Prasad T, Moe O, Dworkin LD, Cutlip DE (2023). Retrieval of renal function after renal artery stenting improves event-free survival in a subgroup analysis of the cardiovascular outcomes in renal atherosclerotic lesions trial. J Vasc Surg.

[24] Miskin N, Qin L, Silverman SG, Shinagare AB (2023). Differentiating benign from malignant cystic renal masses: a feasibility study of computed tomography texture-based machine learning algorithms. J Comput Assist Tomogr.

[25] Ding JL, Xing ZY, Jiang ZX, Chen J, Pan L, Qiu JG (2018). CT-based radiomic model predicts high grade of clear cell renal cell carcinoma. Eur J Radiol.

[26] Zwanenburg A, Vallières M, Abdalah MA, Aerts HJWL, Andrearczyk V, Apte A (2020). The image biomarker standardization initiative: standardized quantitative radiomics for high-throughput image-based phenotyping. Radiology.

[27] Lundberg SM, Nair B, Vavilala MS, Horibe M, Eisses MJ, Adams T (2018). Explainable machine-learning predictions for the prevention of hypoxaemia during surgery. Nat Biomed Eng.

[28] Modrall JG, Jeon-Slaughter H, Ramanan B, Tsai S, Miller RT, Hastings JL (2023). Predicting renal function response to renal artery stenting. J Vasc Surg.

[29] Kashyap VS, Sepulveda RN, Bena JF, Nally JV, Poggio ED, Greenberg RK (2007). The management of renal artery atherosclerosis for renal salvage: does stenting help?. J Vasc Surg.

[30] Simeoni M, Borrelli S, Garofalo C, Fuiano G, Esposito C, Comi A (2021). Atherosclerotic-nephropathy: an updated narrative review. J Nephrol.

[31] Lal H, Singh P, Ponmalai K, Prasad R, Singh SP, Yadav P (2022). Role of blood oxygen level-dependent magnetic resonance imaging in studying renal oxygenation changes in renal artery stenosis. Abdom Radiol.

[32] Hammoud SH, AlZaim I, Al-Dhaheri Y, Eid AH, El-Yazbi AF (2021). Perirenal adipose tissue inflammation: novel insights linking metabolic dysfunction to renal diseases. Front Endocrinol.

[33] Krishnan R, Rajpurkar P, Topol EJ (2022). Self-supervised learning in medicine and healthcare. Nat Biomed Eng.

[34] Dong D, Fang MJ, Tang L, Shan XH, Gao JB, Giganti F (2020). Deep learning radiomic nomogram can predict the number of lymph node metastasis in locally advanced gastric cancer: an international multicenter study. Ann Oncol.

[35] Kottlors J, Bratke G, Rauen P, Kabbasch C, Persigehl T, Schlamann M (2023). Feasibility of differential diagnosis based on imaging patterns using a large language model. Radiology.

[36] Zhou YK, Chia MA, Wagner SK, Ayhan MS, Williamson DJ, Struyven RR (2023). A foundation model for generalizable disease detection from retinal images. Nature.

